# Quality of care for people with multimorbidity – a case series

**DOI:** 10.1186/s12913-017-2724-z

**Published:** 2017-11-18

**Authors:** Michaela L. Schiøtz, Dorte Høst, Mikkel B. Christensen, Helena Domínguez, Yasmin Hamid, Merete Almind, Kim L. Sørensen, Thomas Saxild, Rikke Høgsbro Holm, Anne Frølich

**Affiliations:** 1 Cross-sectoral Research Unit, The Danish Capital Region, Bispebjerg and Frederiksberg Hospital, Copenhagen, Denmark; 2 Research Unit for Chronic Conditions, Bispebjerg and Frederiksberg Hospitals, University of Copenhagen, Copenhagen, Denmark; 3 Department of Clinical Pharmacology, Bispebjerg and Frederiksberg Hospitals, University of Copenhagen, Copenhagen, Denmark; 4 Department of Cardiology, Bispebjerg and Frederiksberg Hospitals, University of Copenhagen, Copenhagen, Denmark; 5 Department of Endocrinology, Bispebjerg and Frederiksberg Hospitals, University of Copenhagen, Copenhagen, Denmark; 6 Department of Respiratory Medicine, Bispebjerg and Frederiksberg Hospitals, University of Copenhagen, Copenhagen, Denmark; 7 General Practice, Frederiksberg, Denmark; 8 General Practice, Copenhagen, Vanløse Denmark; 9 Health Prevention Center, Municipality of Copenhagen, Copenhagen, Denmark; 10 Research Unit for Chronic Conditions, Department of Clinical Epidemiology, Bispebjerg and Frederiksberg Hospitals, University of Copenhagen, Copenhagen, Denmark

**Keywords:** Comorbidity, Delivery of health care – organization and administration, Health care quality, access, and evaluation, Disease management, Medication errors, Qualitative research

## Abstract

**Background:**

Multimorbidity is becoming increasingly prevalent and presents challenges for healthcare providers and systems. Studies examining the relationship between multimorbidity and quality of care report mixed findings. The purpose of this study was to investigate quality of care for people with multimorbidity in the publicly funded healthcare system in Denmark.

**Methods:**

To investigate the quality of care for people with multimorbidity different groups of clinicians from the hospital, general practice and the municipality reviewed records from 23 persons with multimorbidity and discussed them in three focus groups. Before each focus group, clinicians were asked to review patients’ medical records and assess their care by responding to a questionnaire. Medical records from 2013 from hospitals, general practice, and health centers in the local municipality were collected and linked for the 23 patients. Further, two clinical pharmacologists reviewed the appropriateness of medications listed in patient records.

**Results:**

The review of the patients’ records conducted by three groups of clinicians revealed that around half of the patients received adequate care for the single condition which prompted the episode of care such as a hospitalization, a visit to an outpatient clinic or the general practitioner. Further, the care provided to approximately two-thirds of the patients did not take comorbidities into account and insufficiently addressed more diffuse symptoms or problems. The review of the medication lists revealed that the majority of the medication lists contained inappropriate medications and that there were incongruity in medication listed in the primary and secondary care sector. Several barriers for providing high quality care were identified. These included relative short consultation times in general practice and outpatient clinics, lack of care coordinators, and lack of shared IT-system proving an overview of the treatment.

**Conclusions:**

Our findings reveal quality of care deficiencies for people with multimorbidity. Suggestions for care improvement for people with multimorbidity includes formally assigned responsibility for care coordination, a change in the financial incentive structure towards a system rewarding high quality care and care focusing on prevention of disease exacerbation, as well as implementing shared medical record systems.

## Background

Multimorbidity is the coexistence of two or more chronic conditions. Prevalence estimates vary widely, but as many as 50 million Europeans may live with multimorbidity [1]. What is certain is that the prevalence of multimorbidity is rising and increases with age and socioeconomic deprivation [2–4]. In the Capital Region of Denmark 21% of the adult population have two or more chronic conditions [5]. Multimorbidity affects quality of life, ability to work, disability, mortality, and processes of care and has a significant impact on healthcare utilization and costs [6].

The relationship between multimorbidity and quality of care has been examined in recent years. Quality of care for patients with multimorbidity is most appropriately measured by processes, rather than outcomes [7]. Some studies have found that higher comorbidity burden is associated with better quality of care [8, 9], but this relationship has not been consistently demonstrated [10]. Quality of care appears to vary substantially by how time-consuming care processes are, rather than by the complexity or number of conditions [11]. Several studies have documented quality of care deficiencies when patients have discordant or unrelated comorbidities [12]. Each condition with unrelated pathophysiology or requiring different care includes a risk of suboptimal management that include missed diagnoses, inadequate treatment and access, and communication barriers [13]. In addition, treatment for multimorbidity often requires multiple condition-specific medications, resulting in polypharmacy and attendant risks [12–15].

Knowledge about how people with multimorbidity are treated and cared for in the Nordic countries with publicly financed health care systems is limited. The aim of the study was to investigate the quality of care for people with multimorbidity in the Danish Healthcare System.

## Methods

In order to investigate the quality of care for people with multimorbidity different groups of clinicians reviewed records from a selected group of patients with multimorbidity. The group of clinicians discussed the quality of care in three focus group interviews. Further, the appropriateness of the medications of the patients was reviewed by clinical pharmacologists using the Screening Tool to Alert doctors to Right Treatment and Screening Tool of Older Persons’ potentially inappropriate Prescriptions (START-STOPP criteria) and the Medication Appropriateness Index (MAI) [16–18].

### Setting

The Danish Healthcare System (DHS) is a publicly funded healthcare system belonging to the same family of healthcare systems as those of the other Scandinavian countries and the United Kingdom [19, 20]. All registered Danish residents are automatically entitled to health care, which is largely free at the point of use [21]. In the DHS, people with chronic conditions are primarily treated by general practitioners (GPs). The GPs act as gatekeepers with regard to referring patients to hospital and specialist treatment. This means that patients usually start the process of seeking health care by consulting their GP, whose job is to ensure that the patient is offered the necessary treatment but also that the patient is not treated at a higher specialization level than necessary. Patients can also be referred for rehabilitation by the GPs or from specialists at the hospital. Rehabilitation can take place at the prevention centers run by municipalities or at rehabilitation programs at hospitals. GPs and specialists (including private specialists and specialists working at the hospitals) can prescribe medication. The prescribed medication can subsequently be bought at pharmacies [20].

### Design

We used a case series with planned chart review. We obtained medical records from the primary care sector, the hospital sector, and the municipality of Copenhagen for individuals with multimorbidity. We randomly selected patients from the Bispebjerg University Hospital (BUH) administrative system who had: 1) two or more of the most prevalent chronic diseases among the adult population in the Danish Capital Region—cardiovascular disease (ICD-10 code I21.0-I21.9), unstable angina (I20.0), stable angina (I25.1), heart failure (I50), type 1 diabetes (E10.0-E10.9), type 2 diabetes (E11.0-E11.9), chronic obstructive pulmonary disease (COPD, J44.x), and depression (DF33.x) [22]; and 2) at least one outpatient or inpatient encounter at BUH between January 1 and December 31, 2013.

### Population

From the group of randomly selected patients we selected a group of 133 individuals that matched the age and gender distribution of the population with multimorbidity [5]. Each individual received a letter with information about the study and a consent form allowing us to obtain and review their records from hospitals in the Capital Region, their general practitioner (GP), and the Municipality of Copenhagen. Approval was obtained from the Danish Data Protection Agency.

Of the 133 selected individuals, 80 (60%) returned a signed consent form. We reviewed their records to verify that each individual had two or more of the qualifying conditions. We excluded 43 patients as it appeared from their patient records that they did not have one or more of the chronic index conditions. Thirty-seven patients were included in the full record review process. Twenty-five (68%) of these patients had received services from the municipality. We contacted the GPs of all 37 patients to obtain complete medical records; three GPs did not provide access to their patients’ medical records and we were unable to reach 10 GPs. Consequently, 24 medical records with information from hospitals, general practice, and the municipality were available. One patient’s medical record was used for pilot testing the review process, leaving 23 records for review in the study. The characteristics of participating patients are listed in Table 1.

**Table 1:** Patient characteristics

Female gender, n (%)	15 (65)
Mean age in years (range)	68.7 (49–88)
Ethnicity, n (%)	
Ethnic Dane	19 (83)
Other ethnicity	4 (17)
Living situation, n (%)	
Alone	15 (65)
In a nursing home	2 (9)
With a partner or children	6 (26)
Mean number of chronic conditions (range)	4 (2–8)
Mean number of prescribed medications (range)	15 (5–23)
Mean number of bed days (range)	12.7 (0–55)

### Chart review and focus groups

In total three focus group meetings were held in order to conduct the chart reviews and discuss the care processes. At the first two focus group meeting eight patient records were reviewed. Seven patient records were reviewed at the third focus group meeting. Each focus group meeting were attended by a group of six consisting of one general practitioner, two consultant physicians, a nurse from the prevention center at the Municipality of Copenhagen, and a nurse specialist from home care nursing at the Municipality of Copenhagen. In total three consultant physicians (consultants in endocrinology, cardiology, and pulmonology) participated in two meetings each, at which the records of all individuals with diseases within their specialty were reviewed. Three different GPs participated, one at each of the three focus group meetings. Two nurse specialists from home care nursing at the Municipality of Copenhagen participated, one of them in one of the meetings, and the other in two of the meetings. Further, one nurse from the prevention center at the Municipality of Copenhagen participated in all three meetings.

Before each meeting, clinicians were asked to review patients’ medical records and assess the quality of care by responding to a questionnaire (Table 2). Clinicians also received general information about each patient that included age, gender, civil status, diagnoses, lifestyle, places of treatment, patient involvement, information transfer between providers, and the use of telehealth and a graphical overview of each patient’s contact with the healthcare system in 2013.Quality of care was assessed based on the clinicians’ group discussions according to their responses to the questionnaire (Table 2). The group discussions focused on each patient’s care and highlighted quality issues and suggestions about how to improve care quality. The responses from the clinicians were based on clinical guidelines, disease management programs and clinical standards for the relevant chronic conditions. The discussions were facilitated by two of the researchers (M.L.S. and D.H.) and were audio taped and transcribed. All transcribed discussions were coded and categorized inductively using qualitative content analysis [23–25], primarily inspired by Graneheim and Lundman [23] and supported by NVivo 10 software. We focused on the manifest content of data [23–25]. We first read the transcribed interviews in their entirety to obtain a sense of the whole [24, 25]. The conclusions of the discussions were then summarized by the two researchers (M.L.S. and D.H.) and presented for the group of clinicians in order to make sure that the participants agreed with the conclusions.

**Table 2:** Themes and questions in questionnaire assessing care used by reviewing clinicians

Overall theme	Specific theme	Questions
Health care services provided	Has the right treatment been offered?	- Do you assess that the patient has received the right professional treatment?
	Has the patient been offered the needed amount of self-management support?	- Do you assess that the patient has received the necessary self-management support (e.g. support to self-monitoring, conduct lifestyle changes and medication management)
	Has the patient been offered appropriate rehabilitation?	- Do you assess that the patient has received relevant and sufficient rehabilitation services?
	Does the patient receive treatment in the appropriate settings?	- Does the patient receive treatment in the appropriate settings?
	Does the patient receive treatment from the appropriate providers?	- Does the patient receive care from relevant providers?
		- Do you assess that the patients have received the necessary care from specialists?
		- Do you assess that the patient has received the necessary nurse care?
		- Do you assess that the patient has received the necessary care from general practice?
		- Do you assess that the patient has received the necessary home nurse care?
Medical treatment	Is medical treatment appropriate?	Does the patient receive appropriate treatment?
	Does medical treatment take comorbidity into account?	Does the medical treatment take co-morbidity into account?
		Are the prescribed medications relevant taken the full pathological picture into account?
	Are medications missing from treatment?	Are there relevant medication missing?
	Is there a risk of drug-drug interactions?	Is there a risk of drug-drug interaction?
	Can some prescribed medications affect comorbidities?	Can some of the prescribed medications affect the patients’ comorbidities?

### Medication review

Two clinical pharmacologists (physicians with a specialty in clinical pharmacology) also reviewed the 23 patient records. Both clinical pharmacologists reviewed all the 23 cases - one as primary data extractor and one as secondary. Possible differences in interpretation, of which there were few, were solved by discussion until unison. Information about prescribed medications was obtained from the patient records and the medication lists of the patients. Prescribed medications were assessed using the validated Pharmaceutical Care Network Europe (PCNE) drug-related problems classification. PCNE defines a drug-related problem as an event or circumstance that requires medical treatment or unequivocally hinders a desired state of health [20]. Medication appropriateness was assessed using the Screening Tool to Alert doctors to Right Treatment and Screening Tool of Older Persons’ potentially inappropriate Prescriptions (START-STOPP criteria) and the Medication Appropriateness Index (MAI) [16–18]. The START-STOPP criteria comprise a list of 114 indications of potentially inappropriate medication use and clinically significant undertreatment. The list, based on consensus among 19 experts in geriatric pharmacology from 13 European countries, was updated in October 2014 [26]. MAI is a standardized method to assess whether pharmacotherapy prescribed for older individuals is appropriate. It comprises an audit of 10 items including indication, dose, and interactions with other drugs and diseases.

## Results

In general, reviewing clinicians assessed that approximately 50% of patients received adequate treatment for the single condition that prompted an episode of care such as a hospitalization, a visit to an outpatient clinic or the general practitioner. However, the care provided to approximately two-thirds of patients did not take comorbidities into account and insufficiently addressed more diffuse symptoms or problems. Medication lists contained inappropriate medications, and lists in primary and secondary care sectors differed.

### A specialized approach to care

Reviewing clinicians found that, for approximately two-thirds of patients, the care did not take comorbidities into account or address problems such as risk of falls, dyspnea, pain, cognitive impairment, poor nutrition, and mental health issues. One of the reviewing nurses said:

“It seems like the problem is that when something is acute, then the other issues are pushed aside and therefore it is overlooked. It seems like physical exercise earlier on could have been relevant for her due to loss of functional capacity”.

From the discussions of the clinicians based on the records it appeared that for most of the patients, part of the treatment was following the clinical guidelines. However, due to the complexity of the symptom pattern with several chronic conditions sometimes combined with social problems, the reviewing clinicians also identified areas of the medical treatment that was not taken care off for most of the patients. One specialist said:

“…Looking at the entire patient course, I see that what is working is the lung treatment – the management of the COPD works. And the heart rehabilitation works but the mental issues are overseen. (…) Also, the hyperthyroidism is overseen. The GP has data saying that she is hyperthyroid which is why she gets atrial fibrillation which we should have managed…”.

Further, in several of the patients’ records the reviewing clinicians identified that there was confusion related to diagnoses:

“…Unfortunately her medication (for COPD) has not been stopped. She doesn’t have COPD. The new lung function test shows that she is normal, and then nobody tells her to stop taking her medication. I feel sorry for her…”.

From the records the reviewing clinicians also identified that hospital specialists were inattentive to conditions outside their own medical specialty. As one clinician said:

“Some of our medical specialists are working with blinders on. They only look at the lungs – as if the patient had no other organs.”

### Preventive care

With regard to the provision of preventive healthcare, the reviewing clinicians found that appropriate preventive healthcare was lacking for a large part of the patients; for example, most patients did not received counseling related to smoking cessation or nutrition nor assistance and support related to physical activity and excess alcohol use. One of the participating nurses said:

“For dyspnoea, COPD and sleep apnoea he gets appropriate treatment (…). Then there is smoking where the treatment is not ideal”.

Within the hospital setting, the reviewing clinicians described how clinicians’ primary focus on acute care made it difficult to integrate prevention and rehabilitation into care. One reviewing clinician from the hospital described prevention as having vanished from the treatment of somatic illnesses:

“When the patients are here (in the hospital), we don’t think about prevention. I know it’s a quality measure, and I think that maybe the nurses care about it, but not the doctors; I don’t think they care about it.”

In addition, it appears from the clinicians’ review of the patient records, that patients did not always receive mandatory yearly or half-yearly check-ups or the recommended flu vaccine.

### Inappropriate prescribing patterns

During record review, clinical pharmacologists identified that the number of prescribed medications for each patient ranged from 5 to 23. They identified at least one inappropriate medication, as determined by the MAI criteria, on 78% of medication lists (Table 3). The proportion of medications for each patient that were inappropriate ranged from zero to 33%, with a median of 13%. The most frequent inappropriate medications were opioids, long-term use of benzodiazepines and of proton pump inhibitors without indication, and duplications (e.g., psychoactive drugs, such as antipsychotics and antidepressants, inappropriately within the same drug class). The records of patients with prescribed antipsychotics lacked documentation of treatment effectiveness and side effects were monitored insufficiently, if at all. Likewise, the need for continuing treatment was not assessed sufficiently. Insufficient treatment with statins and medications for osteoporosis was common. For 16 (70%) patients, clinical pharmacologists identified discrepancies between medication lists in the primary and secondary care sectors; the number of discrepancies ranged from one to eight. They primarily related to as-needed (PRN) sleep medications, nonsteroidal anti-inflammatory drugs, and morphine.

**Table 3:** Medication review results

Discrepancy between sectors in medication lists, %	70
Medication Appropriateness Index	
Patients with medication lists rated as inappropriate, %	78
Number of inappropriate medications per patient, median (range)	2 (0–6)
Inappropriate medications as percentage of total medications per patient, % (range)	13 (0–33)
START-STOPP	
Number of START-STOPP points per patient, median (range)	2 (0–5)
Number of medications per patient with discrepancy between sectors in medication lists, median (range)	1 (0–8)

Abbreviations: *START-STOPP* screening tool to alert doctors to right treatment and screening tool of older persons’ potentially inappropriate prescriptions

In general, the medication review revealed that the quality of pharmacotherapy varied substantially between patients. The records of some patients reflected adequate treatment, while others reflected too many or too few medications.

### Factors affecting the quality of care for people with multiple chronic conditions

During the focus group discussions the reviewing clinicians identified several factors affecting the quality of care provided to patients with multiple chronic conditions. Relatively short consultation times (10–15 min) in general practice and at hospital outpatient clinics were mentioned several times as a barrier to providing comprehensive care for people with multiple conditions. A specialist described this challenge:

“…preparation time prior to seeing the patients is the key. And there is no preparation time prior to any of the patients. We need to do it directly. (…) This means I need to do overtime work the day before I have outpatient consultations – using my own time to look through the records. I am not always doing that. (…). Alternatively, I use two minutes out of the ten minutes to read what I can from the last two notes, check up the blood tests, check up on the results from the scanning if the person has been scanned, and then when the person enters, I need to update the medication chart, talk to the patient, if I have time to look at the patient at all, how is he and everything, and then when the patient is finished, I need to dictate and close down – in ten minutes total.”

Also, the specialized mentioned focus of care as a barrier for providing high quality care for people with multimorbidity. A physician stated:

“It is a little bit too complicated. With that I think of this patient if it is mental or what it is. Somewhere it is mentioned that he is known with osteoporose, and then he needs a DEXA scanning (…). If he has osteoporosis and he walks around with some micro fractures. Maybe this is actually what is wrong with him when he comes in (to the hospital) with pain. So you can say that you should look at him with new eyes. Who should do that? That is the problem with the specialties that they operate within their own field and focus on that.”

Further, the lack of care coordination was mentioned as a challenge in order to get coordinated high quality care:

“It seems like the patients express a wish for being self-sufficient and be able to get around. She wants to exercise. But it is like nothing is happening. No one picks it up. Then when the nurse from the prevention center thinks she has COPD and is referred to the GP, no one gather the threads and sees whether she gets in contact with the GP and what the result was”.

Reviewing clinicians also identified the need for information technology (IT) tools providing a treatment overview and reminders related to patients’ test results to ensure better and more coordinated management of patients. Such tools were perceived as potentially alleviating healthcare professionals’ overfilled schedules. However, because they were not available health care professionals relied on patients to convey relevant information. When this information was unavailable, health care professionals found it difficult to obtain a treatment overview, and errors resulted. As one reviewing clinician described:

“There are no clear solutions. You just have to prioritize. We have a lot of different tasks – for example, we do regular check-ups of COPD and diabetes patients, and we do it blindfolded so to speak – just because it’s in the guidelines. And that’s really resource-intensive. If we had some tools; for example, a red light flashing could indicate a patient in need of something special (…) if we could prioritize our time differently. Such tools could be created based on our existing databases, and hereby we could actually identify patients at risk, for example, by looking at patients who are often hospitalized”.

Also, the reviewing clinicians noted the difficulty of obtaining a comprehensive and accurate overview of patients’ prescribed medications, which could result in duplicate, contraindicated, or otherwise erroneous prescriptions and medication errors. In relation to this, the clinicians described that updating and managing medication lists was time-consuming, converting hospitalized patients’ medication to the standard hospital formulary was challenging, and distinguishing between different but closely related medications could be difficult. They also pointed out that poor awareness of patients’ comorbidities made it harder to assess the need for medication changes and described trying to assess patients’ medication problems with inadequate information:

“She has pain and this is why she gets in contact with the GP. The GP is lost several times because the medication list is not up-dated. So many of the consultations are just focused on getting the medication right. That is a problem and the patient gets lost in it”.

Additionally, reviewing clinicians described the time-consuming and complicated process of tapering patients off sedative medications. Avoiding the inappropriate continuation of medications required obtaining comprehensive information and scheduling several follow-up visits, both of which were difficult for reviewing clinicians to accomplish.

## Discussion

The current study examined the quality of care for people with multimorbidity by reviewing the records of 23 patients for an entire calendar year and using focus group discussions to elucidate quality issues and important factors contributing to them. In general, care provided to people with multimorbidity focused on treatment for a single disease and did not take into account either comorbidities or more general problems such as pain, mental health issues, dizziness and cognitive impairment. Preventive care was provided to only a limited degree. Record review by clinical pharmacologists identified inappropriate medications in the majority of patients’ medication lists, and discrepancies between medication lists in the primary and secondary care sectors were identified for the majority of patients.

Reviewing clinicians identified several factors contributing to inadequate care: time pressure, lack of comprehensive information about patients, lack of required information technology tools, specialization of care, and neglect of preventive care in the hospital setting. They described medication issues as related to a lack of comprehensive information about patients and time pressures.

Our results are consistent with those of other studies reporting quality of care deficiencies when patients have discordant or unrelated comorbidities [27–31]. Multimorbidity that includes both physical and mental health conditions is associated with a greater risk of quality and safety issues [32]. Physicians are less likely to address diffuse symptoms like pain and breathing problems among patients with poorer health status [33]. Multiple studies in primary care settings suggest that the quality of preventive care is not associated with increasing multimorbidity [8, 34, 35]; however, our assessment of preventive care included secondary care settings and we were unable to identify comparable studies to which we could compare our findings.

Record review by clinical pharmacologists identified that 78% of medication lists had at least one inappropriate drug, according to the MAI criteria. Previous studies report inappropriate medication rates of 15% to 59% at the level of patients [36–38]. Because we examined multiple medication lists for the patients in our study, we cannot directly compare our findings to these results; however, we note that the prevalence of inappropriate medications appears to be relatively high among the patients we studied. Other studies also found that benzodiazepines were a commonly inappropriate medication among patients with multimorbidity [36].

Factors identified by reviewing clinicians as affecting the quality of care were similar to those reported in other studies of care for people with multiple chronic conditions. For instance, lack of time has been identified as a barrier to providing care for patients with multimorbidity [13, 30, 39]. Longer consultations result in more preventive health advice, less prescribing, and increased patient satisfaction [40]. The need in primary care for care management software integrated into patients’ EHRs has also been previously reported [41], and related challenges posed by a lack of adequate patient information are well known [39]. A pervasive and persistent need for systematic medication reconciliation exists across care settings, particularly during transitions between home and hospital care [42].

Strengths of our study include detailed review of medical records for an entire year by clinicians from municipal, general practice, and hospital care settings and a medication review conducted by two clinical pharmacologists. Additionally, the cases were selected based on evidence about combinations of chronic conditions in the larger population, ensuring that the selected combinations of diseases were the most prevalent disease clusters. Study limitations include using medical records as a complete and accurate reflection of care; it is possible that the records we reviewed did not contain documentation of some aspects of care. Further, the way the medical records were obtained entailed a selection process. This meant that the most ill persons with multimorbidity highly probable were not included in the study population as they had died before the data collection started or were too sick to return the consent form. Additionally, the process implied that medical records of patients where we were not able to get in contact with their GP or where the GP refused to share the medical records were not included in the review. This could mean that the medical records we reviewed are not representative for the entire group of GPs. However, our findings are consistent with those of other similar studies, suggesting that our methods detected known patterns of care. Another limitation is the relatively small sample of patients. The study population consisted of individuals living with combinations of specific chronic conditions. Recruiting a broader range of patients with other chronic conditions than the selected could have resulted in more differentiating categories in the focus groups. Further, the study group was selected from Bispebjerg University Hospital. The population in the catchment area of Bispebjerg University Hospital is characterized by having lower educational attainment, a high number of people without a job and lower income than the average in the Capital Region [22]. This could potentially influence the generalization of the results. However, the study population included both people with high- and low educational attainment and as the results showed no difference in the quality of care received by patients from different socio-economic groups, we do not believe that this have impacted the results significantly. Three focus groups meetings were held. Most of the clinicians who participated in the focus groups differed at each meeting and only one person participated in all three meetings. However, having different participants at each meeting could have impacted results e.g. other aspects of how care was provided could have been raised. Additionally, the reviewed medical records are from 2013. Significant changes in the DHS since 2013 includes the introduction of a Shared Medical Card, which allows health care providers from the different health care sectors to access information about patient medication and vaccinations. Whether the Shared Medical Card will improve prescribing has yet to be seen, as it needs to be updated frequently to convey the correct medication list. However, we assume that lack of time to review and access the appropriateness of the medication for people with multimorbidity is still a problem impacting the quality of the medical treatment negatively.

## Conclusions

Our findings indicate that quality issues exist for people in Denmark with multimorbidity that are similar to those reported for other populations. Multiple strategies are required to shift the focus of primary and secondary care from a focus on single diseases to person-centered care, facilitate the availability of patient information to healthcare professionals across care sectors, and prevent potential adverse drug events. Further research is needed to explore methods to improve care for people with multimorbidity.

## Abbreviations

COPD: chronic obstructive pulmonary disease
MAI: Medication Appropriateness Index
NSAIDs: nonsteroidal anti-inflammatory drugs
PCNE: Pharmaceutical Care Network Europe
PRN: pro re nata
START-STOPP criteria: Screening Tool of Older Persons’ potentially inappropriate Prescriptions and Screening Tool to Alert doctors to Right Treatment
